# From Leader Narcissistic Rivalry to Unethical Pro-Organizational Behavior: The Mediating Role of Workplace Ostracism and the Moderating Role of Underdog Expectations

**DOI:** 10.3390/bs16091658

**Published:** 2026-09-16

**Authors:** Sulaymon Jamolidinovich Jabarov, Alisher Tohirovich Dedahanov, Abdulkhamid Komil ugli Fayzullaev, Odiljon Sobirovich Abdurazzakov, Soo Young Shin

**Affiliations:** 1 School of Business, Yeungnam University, Gyeongsan 38541, Republic of Korea; sulaymonjabarov@yu.ac.kr (S.J.J.); swim1@ynu.ac.kr (S.Y.S.); 2 School of Management, New Uzbekistan University, Tashkent 100000, Uzbekistan; a.dedahanov@newuu.uz (A.T.D.); o.abdurazzakov@newuu.uz (O.S.A.)

**Keywords:** leader narcissistic rivalry, narcissistic admiration and rivalry concept, workplace ostracism, underdog expectations, unethical pro-organizational behavior, conservation of resources theory

## Abstract

Unethical pro-organizational behavior (UPB) is gaining scholarly attention due to its harmful consequences, yet limited research explains how employees’ perceptions of leaders’ personality traits drive such behavior. Drawing on conservation of resources theory and the Narcissistic Admiration and Rivalry Concept, this study examined whether and how perceived leader narcissistic rivalry predicts employee UPB. We argue that leaders high in narcissistic rivalry devalue and exclude others, increasing workplace ostracism. Ostracism, in turn, depletes employees’ psychological resources and motivates UPB as a compensatory strategy. We further propose that underdog expectations amplify this effect by shaping employees’ interpretations of exclusion. Using a three-wave, time-lagged survey of 383 full-time employees in South Korea and analyzing data with PROCESS Macro, results show that perceived leader narcissistic rivalry positively predicts workplace ostracism. Workplace ostracism increases UPB and mediates the relationship between leader narcissistic rivalry and UPB. Moreover, underdog expectations strengthen the positive association between ostracism and UPB, with stronger effects among employees high in such expectations. These findings identify workplace ostracism as a key resource-depletion mechanism linking leader narcissistic rivalry to unethical behavior and highlight underdog expectations as an important boundary condition. They highlight the need to manage narcissistic leadership, reduce ostracism, and strengthen ethical norms.

## 1. Introduction

Unethical pro-organizational behavior (UPB) refers to actions intended to benefit an organization or its members that violate core societal values, laws, or standards of conduct (Umphress & Bingham, 2011). While such behavior seeks to help the target organization, it remains unethical. However, UPB is widespread in Western (e.g., the United States and Europe) and Eastern contexts (e.g., China) (Y. Wang et al., 2022; Yao et al., 2022). High-profile cases illustrate the consequences of this type of behavior. Volkswagen employees in Germany manipulated software to evade environmental regulations (Castille et al., 2018), and Boeing employees concealed safety issues with the 737 Max program that contributed to fatal crashes (Chen et al., 2023). Nike in China faced accusations of misrepresenting product features (X. Zhang et al., 2020). UPB can produce severe harm to individuals, organizations, and society as a whole. As a result, scholars have intensified efforts to identify the antecedents of this behavior.

Among such antecedents, leadership has emerged as a pivotal influence on employee UPB. Earlier studies have predominantly examined the effects on UPB of various styles of leadership including ethical, transformational, empowering, charismatic, and authoritarian leadership, as well as abusive supervision (R. Ahmad et al., 2024; Mishra et al., 2022), but considerably less attention has been paid to the personal characteristics of leaders. A recent review of the literature on UPB highlights the necessity of exploring leaders’ personality traits as an important but underexplored predictor of UPB (R. Ahmad et al., 2024). A significant gap remains with respect to how leaders’ dispositional traits influence employees’ UPB. This distinction is crucial: while destructive leadership constructs such as abusive supervision can capture specific behavioral patterns, personality traits provide a deeper lens into fundamental leader behaviors and their effects on followers (Gauglitz et al., 2023). In particular, narcissistic rivalry, which is a dimension of narcissism that is characterized by antagonistic self-protection, hostility, and the devaluation of others (Back et al., 2013), might be particularly salient for the understanding of employees’ unethical behavior that is intended to benefit an organization or its members, including its leaders, as its core motivations revolve around the preservation of superiority and self-interest. While recent evidence suggests that narcissistic leadership can increase employee UPB (Queiri et al., 2026), the treatment of narcissism as a unidimensional construct allows a critical gap in understanding with respect to which specific facets of leader narcissism foster employee UPB.

The Narcissistic Admiration and Rivalry Concept (NARC) (Back et al., 2013) differentiates between narcissistic admiration, involving assertive self-enhancement and charm, and narcissistic rivalry, marked by antagonism and the devaluation of others. In support of this distinction, scholars who investigate employees’ narcissistic tendencies show that narcissistic rivalry is more strongly linked to UPB than narcissistic admiration (Grabowski et al., 2025). Nevertheless, it remains uncertain whether and through what mechanisms leaders’ narcissistic rivalry could similarly promote UPB among subordinates. Focusing on narcissistic rivalry, this study advances UPB research, going beyond the examination of destructive leadership behaviors and providing a more nuanced understanding of how specific facets of leader narcissism influence employees’ unethical behavior.

Leaders high in narcissistic rivalry often act in hostile and antagonistic ways toward their subordinates (Breevaart & Schyns, 2025; Gauglitz et al., 2023), which can lead to these employees feeling excluded or ignored in the workplace (Howard et al., 2020; Özkan, 2022; Z. Wang et al., 2021). Prior research shows that workplace ostracism, which entails individuals’ perception of being ignored or excluded by others at work, increases the likelihood of engaging in UPB (S. Zhang, 2019). However, the underlying mechanisms that link leader narcissistic rivalry to UPB remain underexplored, and this leaves an important gap in the literature. How does leaders’ narcissistic rivalry lead employees to engage in unethical acts to benefit the organization? We address this question with the help of the conservation of resources (COR) theory (Hobfoll et al., 2018), which posits that individuals strive to obtain, protect, and restore valued resources. Among these is the critical relational resource of social inclusion. Employees who experience ostracism lose this resource. This loss produces psychological strain, motivating compensatory behavior. Employees may engage in UPB to regain acceptance, restore their status, or signal their loyalty to the organization. Workplace ostracism therefore functions as a resource-depletion mechanism that links leader narcissistic rivalry to UPB.

Further, this process is unlikely to affect all employees equally. Individual differences shape the ways that employees interpret and respond to exclusion. We focus on underdog expectations—defined as the perception that others have underestimated one’s capabilities or potential (Nurmohamed, 2020). Employees with high underdog expectations often feel a strong need to prove others wrong (Xue et al., 2022). This motivation can push them toward extreme and risky behaviors, including unethical actions (Loi et al., 2022). When such employees experience ostracism, the pressure on them intensifies. They might engage in UPB to restore their image and demonstrate their value to the organization. In this way, underdog expectations tend to amplify the ostracism–UPB relationship. In spite of its potential significance, this moderating effect has not yet been empirically examined, which allows for an important gap in boundary conditions regarding cases in which workplace ostracism translates into UPB. Thus, we test whether the positive relationship between workplace ostracism and UPB becomes stronger at higher levels of underdog expectations.

Responding to the research gaps identified above, this study develops and tests a model that is grounded in COR theory linking leaders’ narcissistic rivalry to employees’ UPB through workplace ostracism, with underdog expectations as a moderator. This study makes three key contributions. First, it advances leadership research, moving beyond unidimensional views of narcissism, focusing on narcissistic rivalry as a distinct and consequential trait. Second, workplace ostracism is identified as a novel mediating mechanism that extends explanations of UPB that go beyond social exchange frameworks. Third, it introduces underdog expectations as a critical boundary condition, indicating when and in whom ostracism is most likely to produce unethical behavior. Together, these contributions integrate leadership traits, resource-based theory, and individual differences to provide a more comprehensive account of UPB.

## 2. Literature Review and Hypothesis Development

### 2.1. Unethical Pro-Organizational Behavior

UPB refers to intentional actions benefiting an organization or its members that violate societal norms, ethical standards, or laws (Umphress & Bingham, 2011). This definition highlights two core features, namely, unethical conduct and pro-organizational intent. UPB is inherently paradoxical. In it, employees act to support their organization, but they do so by breaching ethical boundaries. Employees often justify their actions by claiming that UPB serves a higher organizational purpose (Mishra et al., 2022; Mo et al., 2023). However, this justification does not reduce its unethical nature. Instead, UPB reflects a tension between moral standards and organizational loyalty. Within this tension, employees can rationalize misconduct as necessary for success.

Umphress and Bingham (2011) clarify that not all unethical behavior can qualify as UPB. Actions that are driven purely by self-interest fall outside this definition. Pro-self unethical behavior benefits the individual alone. By contrast, UPB benefits the organization or both the organization and the individual (Mishra et al., 2022). UPB could involve acts of commission, such as exaggerating performance, or acts of omission, such as withholding critical information (Umphress et al., 2010). Such acts are also intentional. Employees knowingly engage in these behaviors to advance the organization’s goals, and this feature distinguishes UPB from errors or negligence.

Despite its intended benefits, UPB rarely produces sustainable positive outcomes (Dadaboyev et al., 2025). While it can generate short-term gains, such as improved performance or competitive gains, these often come at a cost. Over time, UPB can lead to reputational damage, legal penalties, and financial loss (Mishra et al., 2022). Bribery, misrepresentation, and similar practices can initially secure opportunities, but they expose organizations to serious risk. Thus, unlike organizational citizenship behavior, UPB ultimately undermines organizational well-being despite its pro-organizational intent (Umphress et al., 2010).

### 2.2. Narcissism and Leadership

Narcissism involves an inflated view of the self, weak interpersonal empathy, and self-regulatory strategies prioritizing personal gain over others’ well-being (Bernerth, 2022). Narcissistic individuals often appear confident, dominant, and socially skilled. They also resist criticism, display entitlement, and exploit others (Hoffman et al., 2013). These traits tend to make narcissism highly relevant in leadership contexts. Individuals who are high in narcissism are strongly driven to seek status and power (Grapsas et al., 2020). They often pursue and attain leadership roles (Gauglitz et al., 2023). However, the findings on narcissistic leadership are mixed. Some studies identify a bright side, linking narcissism to charisma and bold decision-making (Braun, 2017; Zhou et al., 2019). However, others emphasize its dark side, including related unethical conduct, relational harm, and abusive supervision (Bernerth, 2022; Ellen et al., 2019; Tiwari & Jha, 2022).

These inconsistencies stem in part from treating narcissism as a unidimensional construct. Taking this approach obscures the meaningful differences that appear across its components (Fehn & Schütz, 2021). To address this limitation, a recent study adopted NARC (Back et al., 2013), which distinguishes two dimensions of narcissism. Narcissistic admiration involves the nature of assertive self-promotion through charm, confidence, and social dominance. In contrast, narcissistic rivalry reflects defensive self-protection, marked by hostility, devaluation of others, and antagonism (Leckelt et al., 2019). These dimensions produce distinct trajectories and outcomes. The positive effects of admiration tend to diminish over time, but the negative effects of rivalry often intensify (Fehn & Schütz, 2021; Leckelt et al., 2015). Thus, a multidimensional perspective would offer a more precise account of narcissistic leadership.

Leader narcissistic rivalry captures the antagonistic and conflict-prone side of narcissism. Leaders who are high in rivalry tend to behave in hostile and demeaning ways toward their subordinates (Gauglitz et al., 2023; Gauglitz & Schyns, 2024). This behavior erodes trust, weakens perceived supervisor support, and damages the quality of relationships (Fehn & Schütz, 2021). Over time, they can also signal rejection and exclusion. Such patterns suggest a clear pathway. When leaders devalue and antagonize their subordinates, employees may feel ignored, excluded, or socially rejected. This aligns with workplace ostracism. As leader behavior shapes the relational climate and ethical norms, narcissistic rivalry could easily trigger downstream behavioral consequences. Therefore, we focus on leader narcissistic rivalry as a key antecedent for workplace ostracism and UPB.

### 2.3. Leader Narcissistic Rivalry and Workplace Ostracism

Narcissistic rivalry is a defensive interpersonal strategy that is marked by efforts to assert dominance and to devalue others. It often manifests as hostility and aggression (Gauglitz et al., 2023). Individuals who are high in narcissistic rivalry are highly sensitive to status threats and respond aggressively to protect their grandiose self-views (Grapsas et al., 2020). When they are in a leadership role, this defensiveness can target their subordinates, such that leaders display antagonistic and demeaning behaviors that can constitute interpersonal mistreatment. Such behaviors threaten employees’ socioemotional resources, including those of belongingness, self-worth, and perceived control.

COR theory indicates that individuals strive to obtain, retain, and protect valued resources, including their social support and positive workplace relationships (Hobfoll et al., 2018). Such interpersonal resources are closely linked with employees’ core needs for belongingness. Leaders who exhibit high levels of narcissistic rivalry can threaten such resources by providing displays of hostility, antagonism, and the devaluation of others. These behaviors not only directly undermine employees’ sense of social worth but may also signal to others that the targeted employee has a lower relational standing in the workplace. Earlier research suggests that, when supervisors engage in exclusionary or hostile actions, colleagues can distance themselves from the victim or even imitate the exclusionary conduct, reinforcing the broader perceptions of exclusion (Howard et al., 2020). Consequently, employees subject to rivalry may experience both a loss of interpersonal resources and an unmet need for belongingness, which together can manifest as heightened perceptions of workplace ostracism (Özkan, 2022; Z. Wang et al., 2021).

Thus, hostile or demeaning treatment may not only harm immediate relationships but also serve as a signal of declining relational standing and potential future exclusion (Ferris et al., 2017; Wu et al., 2015). Over time, these signals accumulate to create a climate of psychological distance. Employees who are working in these environments become more vigilant to cues of exclusion and are more likely to interpret interactions as ostracizing. Thus, leaders who are high in narcissistic rivalry foster sustained interpersonal climates, heightening perceptions of workplace ostracism. Thus, we propose:

**Hypothesis 1.**  *Leader narcissistic rivalry is positively associated with workplace ostracism.*

### 2.4. Workplace Ostracism and Unethical Pro-Organizational Behavior

Workplace ostracism threatens fundamental psychological needs, including those of belongingness, self-esteem, control, and meaningful existence (M. Li et al., 2021). Earlier research has shown that ostracism could trigger mixed behavioral responses (Mao et al., 2017). Some individuals may produce antisocial behaviors such as aggression and hostility to reassert control (Ren et al., 2018). Others can respond with prosocial behaviors such as helping to regain acceptance (Bedi, 2021).

These responses do not capture the full range of reactions. Ostracized employees can also engage in UPB, a response that remains underexplored. COR theory provides a useful lens. Workplace ostracism can deplete employees’ psychological and social resources (H. Zhu et al., 2017). When individuals confront resource loss, they are motivated to quickly recover those resources (Hobfoll et al., 2018). Resource loss can trigger risky or norm-violating behavior that is aimed at restoring the resource base. Here, UPB serves as a strategic, albeit unethical, response to ostracism. Employees who feel excluded can lose critical socioemotional resources. To compensate for this, they can engage in behaviors to signal loyalty, increase perceived value, and restore social standing. UPB fits this function. While it violates ethical standards, it can help employees regain approval and reestablish their place within the organization.

Moreover, UPB is inherently goal-directed. It aims to benefit the organization or both the organization and the self (Mishra et al., 2022). For ostracized employees, this dual function is critical. Through engagement in UPB, they simultaneously support organizational goals and address their need for inclusion and validation. Thus, UPB is a high-risk but potentially effective strategy for the restoration of one’s resources.

This dynamic could be particularly relevant in countries like South Korea. In these contexts, individuals tend to assign high value to group harmony, belonging, and social acceptance (Park & Han, 2018). Employees can depend heavily on group membership and are likely to be motivated to maintain their standing within it (Huwaë & Schaafsma, 2019). When ostracism threatens this standing, the pressure on employees to restore their inclusion intensifies. These employees could therefore prioritize organizational interests even at an ethical cost to regain acceptance. Earlier studies support this argument, finding that ostracized employees engage in UPB to signal value and to restore belonging (Thau et al., 2015; S. Zhang, 2019).

Taken together, these arguments suggest that workplace ostracism increases employees’ motivation to engage in UPB to restore lost social resources and organizational acceptance. Accordingly, we propose:

**Hypothesis 2.**  *Workplace ostracism is positively associated with unethical pro-organizational behavior.*

### 2.5. The Mediating Role of Workplace Ostracism

Previous studies have shown that leader narcissism predicts unethical leadership behaviors (Blair et al., 2017; Mitchell et al., 2023) and increases employees’ engagement in UPB (Queiri et al., 2026). Among its dimensions, narcissistic rivalry marked by antagonism, hostility, and a drive to dominate others appears particularly harmful (Gauglitz et al., 2023; Gauglitz & Schyns, 2024). Studies have further indicated that unethical leader behavior often cascades downward, shaping subordinates’ unethical actions (Lian et al., 2022; Mesdaghinia et al., 2023). However, the underlying mechanism here remains unclear. How does a leader’s narcissistic rivalry translate into employee UPB?

Drawing on COR theory (Hobfoll et al., 2018), we argue that workplace ostracism can explain this link. Leaders who are high in narcissistic rivalry tend to damage relationships, reduce engagement, and display abusive behaviors (Fehn & Schütz, 2021; Gauglitz et al., 2023). Because leaders have formal authority, their antagonistic actions signal devaluation and exclusion. These signals encourage psychological distance, and they create a climate of ostracism (Z. Wang et al., 2021). Subordinates may perceive that interacting with ostracized employees could lead to their being ostracized. Consequently, they could also engage in ostracism as a result of concerns regarding the risk of not excluding the individual (Howard et al., 2020). Employees who perceive this exclusion experience a loss of critical socioemotional resources, including their sense of belonging and support.

This resource loss has behavioral consequences. COR theory posits that individuals seek to protect and restore depleted resources. When they are ostracized, employees’ belongingness and social standing are threatened. They respond by engaging in behaviors to restore their acceptance and signal value (Imboden, 2024). Some can adopt prosocial strategies to repair their relationships (Haldorai et al., 2022). Others, by contrast, can pursue self-enhancement through ingratiation or impression management (H. Zhang et al., 2025). However, these strategies can take unethical forms. Employees might engage in UPB as a calculated resource investment that signals loyalty, enhancing their perceived usefulness and regaining their inclusion. This tendency becomes more pronounced in collectivistic contexts, where employees prioritize group goals and social harmony. The empirical evidence supports this argument: ostracized employees tend to engage in UPB to improve their inclusionary status (Thau et al., 2015; S. Zhang, 2019).

Taken together, workplace ostracism appears to function as a resource-depletion mechanism linking leader narcissistic rivalry to employee UPB. Leader narcissistic rivalry increases perceived ostracism, which in turn motivates employees to engage in UPB as a strategy for resource restoration. Accordingly, we propose:

**Hypothesis 3.**  *Workplace ostracism mediates the relationship between leader narcissistic rivalry and unethical pro-organizational behavior.*

### 2.6. Moderating the Role of Underdog Expectations

The preceding arguments suggest that workplace ostracism increases UPB, depleting employees’ resources and triggering compensatory behavior. But this effect should not be uniform. Prior studies have shown that the consequences of ostracism depend on contextual and individual factors (Asmita et al., 2025; Mao et al., 2017). We therefore examine underdog expectations as a key moderator. Underdog expectations are employees’ perceptions that others underestimate their abilities or potential (Nurmohamed, 2020).

These perceptions indicate the ways that employees interpret adversity and guide their behavioral responses. When individuals perceive low expectations, they tend to respond with increased effort and persistence to disprove these judgments (Dai et al., 2018; Vega et al., 2015). However, this motivation has a darker side. The drive to prove others wrong can push individuals to perform risky or unethical strategies when such actions can promise effectiveness. Under conditions of workplace ostracism, this dynamic only intensifies. Ostracism signals rejection and reduces access to social resources. According to COR theory (Hobfoll et al., 2018), underdog expectations can threaten critical resources, including status, recognition, and future opportunities, especially when it is combined with exclusion. When employees perceive that these resources are endangered or exhausted, the desperation principle of COR theory suggests that they may adopt defensive and sometimes aggressive or irrational behaviors to protect themselves. Consequently, employees with high underdog expectations can interpret exclusion as both a threat and a challenge. This perception can fuel a stronger need to restore their status, demonstrate their competence, and gain recognition. In this context, heightened motivation in this context increases the likelihood that an employee will engage in UPB if it appears to be instrumental for them to regain acceptance and signal value. Empirical evidence supports this claim: underdog expectations can promote unethical behavior (Z. Li et al., 2026; Loi et al., 2022).

**Figure 1:**
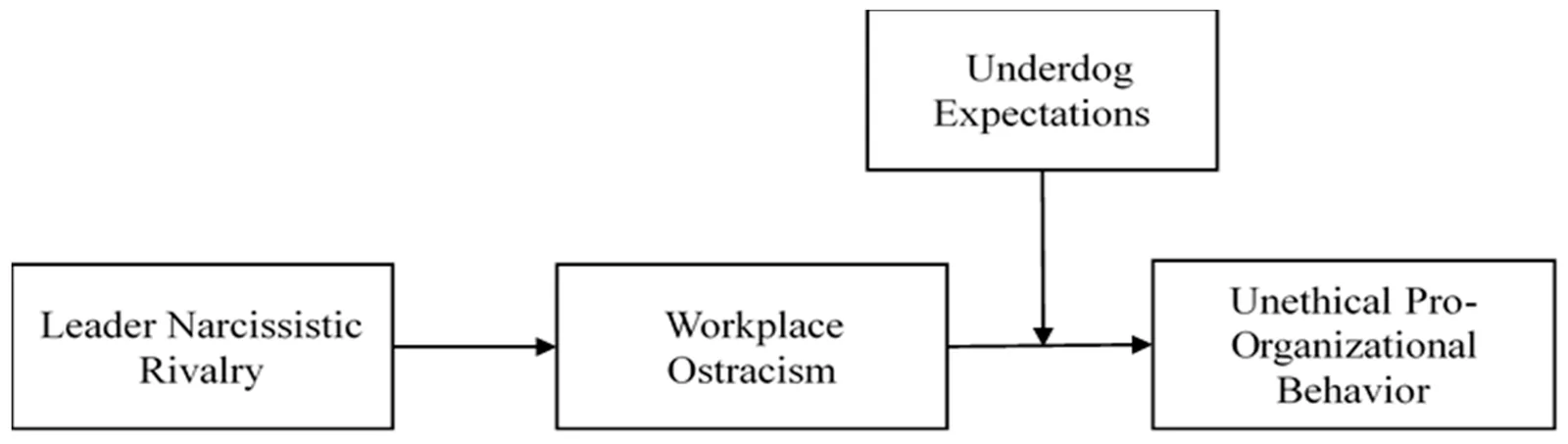
Proposed research model.

In contrast, employees who have low underdog expectations face less pressure to prove themselves. For these individuals, ostracism could still deplete resources, but this generates less urgency to engage in unethical compensatory behaviors. As a result, the link between ostracism and UPB should be weaker. Following COR theory, we argue that underdog expectations amplify the effect of workplace ostracism on UPB by intensifying perceived resource threat and motivating stronger resource recovery efforts. Accordingly, we propose (Figure 1):

**Hypothesis 4.**  *Underdog expectations moderate the positive relationship between workplace ostracism and unethical pro-organizational behavior, such that the relationship is stronger when underdog expectations are high rather than low.*

## 3. Methods

### 3.1. Sample and Procedure

The sample consisted of full-time employees working in manufacturing companies located in Gyeongbuk Province and Daegu Metropolitan City, Republic of Korea. Manufacturing companies were selected because they are central to the country’s economy and its exports (Xu & Sim, 2018); in addition, several prominent cases of UPB have been documented in this sector, such as in the companies Volkswagen and Boeing (Castille et al., 2018; Chen et al., 2023). Moreover, full-time employees were selected because they are more likely to have sustained interactions with their supervisors and coworkers, enabling informed assessments of the focal constructs (Queiri et al., 2026; Y. Zhu et al., 2023). To facilitate access to potential participants, we contacted human resource managers, explained the study’s purpose and practical relevance, and obtained their approval. After their support was obtained, employees completed a paper-and-pencil survey administered on site. A convenience sampling approach was used due to access and confidentiality constraints, which are common in organizational studies (S. Ahmad et al., 2025; Gao et al., 2026). Data collection across multiple organizations increases contextual heterogeneity, alleviating concerns regarding sample representativeness (Mehta et al., 2019).

Because our focal constructs were leader narcissistic rivalry, workplace ostracism, underdog expectations, and UPB, reflecting subjective perceptions, we used self-report measures. Employees are best positioned to report their experiences. Prior studies also showed that self-reports are the dominant approach for assessing UPB, given its covert nature (R. Ahmad et al., 2024; Umphress et al., 2010). Subordinate ratings also provide valid assessments of leader narcissism (Fehn & Schütz, 2021). Thus, the self-report measurement approach aligns with established practice.

To mitigate common method bias, we adopted a three-wave, time-lagged design (Podsakoff et al., 2024). The waves were separated by one month, consistent with previous studies on workplace ostracism and UPB (Z. Wang et al., 2021; Yao et al., 2022). Each survey was prefaced by a cover letter that explained the study’s purpose. The participants were assured that their participation was voluntary and anonymous, that their responses would remain confidential and accessible only to the research team, and that the data would be used solely for research purposes. We also emphasized that there were no right or wrong answers, and we encouraged honest responses.

To determine the size of the target sample, we followed a sample-to-item ratio of 20:1 (Islam et al., 2020) and invited 650 employees to participate. At Time 1, the employees rated their leaders’ narcissistic rivalry and reported their underdog expectations. We also measured leader narcissistic admiration (as a control variable) and collected demographic information. A total of 552 questionnaires were returned for a response rate of 84.92%. At Time 2, we recontacted these participants and collected data on workplace ostracism. At this stage, 430 questionnaires were returned for a response rate of 77.90%. At Time 3, we measured UPB and obtained 395 responses for a response rate of 91.86%. The responses across waves were matched with the use of unique participant codes, and incomplete responses were removed. The final sample comprised 383 valid responses, resulting in an overall response rate of 58.92%, which is comparable to those reported in prior multi-wave organizational studies (Z. Wang et al., 2021; S. Zhang, 2019).

The mean respondent age was 38.07 years (SD = 11.31), and 36% were female; 65.5% held a bachelor’s degree, 30.5% a master’s degree, and 3.9% a doctoral degree. The tenure distribution was as follows: 14.4% less than 1 year, 30.5% between 1 and 4 years, 31.1% for 5–9 years, 21.7% for 10–15 years, and 2.3% more than 15 years.

### 3.2. Measures

Because the original scales were developed in English, rigorous translation and back-translation procedures were implemented to ensure linguistic accuracy. Unless otherwise noted, all items used a five-point Likert scale (1 = strongly disagree, 5 = strongly agree).

Leader Narcissistic Rivalry was measured using three items from the Narcissistic Admiration and Rivalry Questionnaire (NARQ; Leckelt et al., 2018). A sample item is as follows: “My leader reacts annoyed if another person steals the show from him/her.” Cronbach’s α = 0.902.

Workplace Ostracism was measured using 10 items from Ferris et al. (2008), rated on a five-point frequency scale (1 = never, 5 = always). A sample item is as follows: “I involuntarily sat alone in a crowded lunchroom at work.” Cronbach’s α = 0.966.

Underdog Expectations were assessed using a three-item scale developed by Nurmohamed (2020). A sample item is “I am viewed as an underdog in doing this job by others.” Because the term underdog may not be universally understood, we provided a brief definition, indicating that it refers to the perception that others consider one to be less capable or less likely to succeed. This step aimed to improve construct clarity and reduce potential misinterpretation. Cronbach’s α = 0.895.

UPB was measured using the six-item scale developed by Umphress et al. (2010). A sample item is as follows: “If needed, I would conceal information from the public that could be damaging to my organization.” Cronbach’s α = 0.942.

Control Variables. Consistent with prior research (Gauglitz et al., 2023; Wurst et al., 2017), we controlled for leader narcissistic admiration to isolate the unique effects of narcissistic rivalry, as these two dimensions are moderately correlated (Back et al., 2013). We measured narcissistic admiration with three items from the NARQ (Leckelt et al., 2018); Cronbach’s α = 0.801). We also controlled for demographic variables, including sex, age, education, and tenure, given their potential influence on unethical behavior (Thau et al., 2015; X. Wang et al., 2022).

## 4. Results

### 4.1. Confirmatory Factor Analysis

A confirmatory factor analysis (CFA) was conducted in AMOS 23 to evaluate the measurement model. The proposed four-factor model of leader narcissistic rivalry, workplace ostracism, underdog expectations, and UPB fit the data well (χ^2^ = 469.191, df = 203, χ^2^/df = 2.311, CFI = 0.966, TLI = 0.962, RMSEA = 0.059). All of the standardized factor loadings exceeded 0.60. Composite reliability values were above 0.70, and average variance extracted (AVE) values exceeded 0.50. These results support convergent validity. In addition, the square root of AVE for each construct exceeded its correlations with other constructs, which supported discriminant validity (Voorhees et al., 2016). We further tested alternative models. Model fit declined substantially when factors were combined (Table 1). This pattern strengthens the confidence level of the distinctiveness of the four constructs.

### 4.2. Common Method Bias

We used multiple approaches to assess common method bias (CMB). First, Harman’s single-factor test was performed and showed that the first factor accounted for 36.42% of the variance, below the 50% threshold (Podsakoff et al., 2024). This result indicates that no single factor dominated the data. Second, the single-factor CFA model showed poor fit (χ^2^ = 3817.633, df = 209, χ^2^/df = 18.266, CFI = 0.543, TLI = 0.495, RMSEA = 0.213), especially when they were compared to the four-factor model (Table 1). Third, we added an unmeasured latent common method factor to the baseline model (Collier, 2020). This addition did not improve the model fit. The chi-square difference was not significant (Δχ^2^ = 1.97, Δdf = 2, *p* > 0.05), and changes in CFI, TLI, and RMSEA were negligible. The factor loadings also remained stable, with a maximum change of 0.049. Finally, variance inflation factor values ranged from 1.040 to 1.076, well below the conservative threshold of 3.0 (Black et al., 2010). Taken together, these results indicate that CMB is unlikely to threaten the validity of the findings.

### 4.3. Descriptive Statistics and Correlations

Before the hypothesis testing, we examined descriptive statistics and bivariate correlations across all variables. Table 2 reports the results. Leader narcissistic rivalry was positively correlated with workplace ostracism (*r* = 0.215, *p* < 0.01) and UPB (*r* = 0.103, *p* < 0.05). Workplace ostracism was positively correlated with UPB (*r* = 0.194, *p* < 0.01). In addition, underdog expectations presented a positive association with UPB (*r* = 0.123, *p* < 0.05). These correlations are in alignment with the hypothesized relationships and provide preliminary support for the proposed model.

### 4.4. Hypothesis Testing

We tested the proposed mediation and moderation effects using the PROCESS macro (Version 4.2) in SPSS 23. Specifically, we used Model 4 to test mediation and Model 1 to test moderation, with 5000 bootstrap resamples and 95% bias-corrected confidence intervals. We selected PROCESS because it efficiently estimates conditional process models and provides clear tests of direct and indirect effects without reliance on the stronger assumptions required by structural equation modeling (Hayes, 2017; M. Yang et al., 2025).

Table 3 reports the regression results. After controlling for all covariates, leader narcissistic rivalry significantly predicted workplace ostracism (β = 0.165, SE = 0.080, *p* < 0.05), supporting H1. Workplace ostracism, in turn, significantly predicted UPB (β = 0.184, SE = 0.053, *p* < 0.001), supporting H2.

We next tested whether workplace ostracism mediated the relationship between leader narcissistic rivalry and UPB. As shown in Table 4, the indirect effect was significant (β = 0.030, SE = 0.017, 95% CI [0.003, 0.069]). After including workplace ostracism, the direct effect of leader narcissistic rivalry on UPB was nonsignificant (β = 0.059, SE = 0.083, 95% CI [−0.103, 0.221]). These findings supported full mediation and provide support for H3.

We then tested whether underdog expectations moderated the relationship between workplace ostracism and UPB. Following Aiken and West (1991), we mean-centered the predictor and moderator variables using the PROCESS defaults. Table 5 shows that the interaction between workplace ostracism and underdog expectations was positive and significant (β = 0.146, SE = 0.041, 95% CI [0.067, 0.225]), supporting H4.

**Figure 2:**
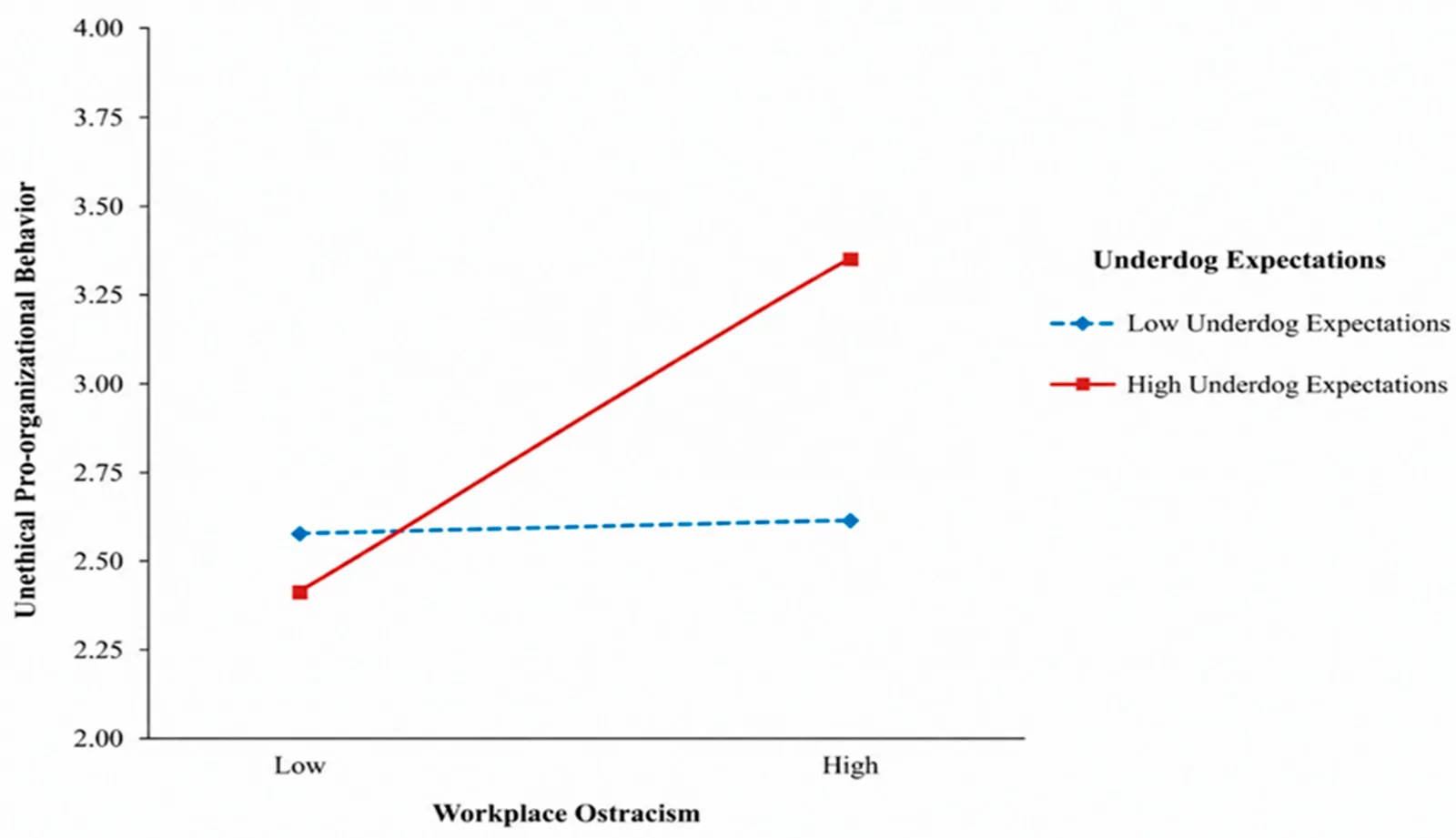
Moderating effect of underdog expectations.

To interpret the interaction, a simple slope analysis was conducted (Aiken & West, 1991). We examined the relationship between workplace ostracism and UPB at low (−1 SD) and high (+1 SD) levels of underdog expectations (Figure 2). At low levels, workplace ostracism does not significantly predict UPB (b = 0.015, SE = 0.069, 95% CI [−0.121, 0.151]), which indicates that its effect on UPB is effectively neutralized. At a high level, the relationship was positive and significant (b = 0.381, SE = 0.076, 95% CI [0.232, 0.529]), which suggests that employees with greater underdog expectations are more likely to respond to workplace ostracism by engaging in UPB. These findings confirm that underdog expectations strengthen the positive effects of workplace ostracism on UPB, providing further support for H4.

## 5. Discussion

Despite extensive study of UPB, the role of leader narcissistic rivalry in UPB remains underexplored. Drawing on COR theory, this study examined whether workplace ostracism would mediate this relationship and whether underdog expectations condition it. Overall, the findings support the proposed model.

The results show that leader narcissistic rivalry positively predicts employees’ perceptions of workplace ostracism. Hitherto, direct evidence on this link has been limited. However, our findings align with prior studies showing that antagonistic and hostile leadership encourages exclusionary, demeaning social environments (Howard et al., 2020; Shafqat et al., 2025). Leaders who are high in narcissistic rivalry tend to devalue others and signal rejection, increasing employees’ sense of exclusion. We also found that workplace ostracism can increase UPB. Earlier studies have primarily associated ostracism with counterproductive and unethical behaviors that are detrimental to organizations (Bedi, 2021; M. Li et al., 2021; Qi et al., 2020), our results indicate that ostracism can also motivate the practice of unethical acts intended to benefit the organization. This aligns with previous findings indicating a positive association between workplace ostracism and pro-group or pro-organizational behaviors (Thau et al., 2015; S. Zhang, 2019). However, unlike earlier research, our work interprets this relationship with a resource-based lens, suggesting that UPB could function as a means of regaining resources threatened by workplace ostracism.

Mediation analysis confirms that workplace ostracism is a mechanism that links leader narcissistic rivalry to UPB. In effect, leader traits seem to contribute to a resource-loss process. Leaders who are high in narcissistic rivalry, through antagonistic actions, signal devaluation and exclusion, foster psychological distance, and create an environment of ostracism in the workplace. This perception of workplace ostracism depletes employees’ socioemotional resources and motivates compensatory behaviors. Employees may then attempt to restore threatened resources through engaging in UPB to demonstrate their value and their commitment to the organization’s objectives. By contributing to organizational interests, employees seek to regain legitimacy and recognition in the workplace. Because leaders often control access to critical organizational resources and evaluations, actions that benefit the organization can also indirectly enhance employees’ standing with their leaders. Thus, UPB may serve as a resource-restoration strategy, providing an indirect means of recovering resources threatened by workplace ostracism. These findings indicate that interpersonal dynamics represent one possible mechanism through which leaders’ personalities can indirectly influence employee unethical conduct. While prior research on leadership and UPB primarily concentrates on mechanisms such as moral disengagement, felt obligation, and organizational identification (Farasat & Azam, 2022; Shaw et al., 2020; Y. Zhang et al., 2020), our results, from a resource-based perspective, add to the literature by highlighting workplace ostracism as an additional relational pathway that links leader characteristics to employee UPB.

Our findings also support the moderating role of underdog expectations. Specifically, the positive relationship identified between workplace ostracism and UPB was stronger when the underdog expectations were high but was nonsignificant when they were low. This pattern aligns with the desperation principle of COR theory, suggesting that individuals facing resource exhaustion can adopt risky strategies to restore resources. Employees who perceive underdog expectations already face threats to critical resources, such as status, recognition, and social standing. When this is combined with workplace ostracism, these threats can intensify the motivation to regain approval, demonstrate competence, and restore one’s standing, even using unethical means. In contrast, employees with low underdog expectations may still perceive ostracism as resource-depleting, but the threat may not be sufficient to trigger such desperation-driven responses, making them less likely to engage in UPB. These findings extend prior research showing that underdog expectations can encourage unethical behaviors, such as employee expediency and cheating (Z. Li et al., 2026; Loi et al., 2022). However, prior studies have largely focused on self-serving unethical conduct that undermines organizational goals. Our findings suggest that resource-depleted employees could also engage in unethical behaviors intended to benefit the organization.

### 5.1. Theoretical Implications

This study advances the study of leader narcissism and UPB in three key ways. First, it goes beyond any unidimensional view of narcissistic leadership by isolating narcissistic rivalry. Prior work treats narcissism as a single construct that is linked to UPB (Queiri et al., 2026). However, this approach masks important differences between its dimensions. Our findings show that the antagonistic aspect of narcissistic rivalry has a central role in driving unethical employee behavior. This finding refines current theory by identifying the aspects of narcissism that matter most.

Second, this study identifies workplace ostracism as a critical mediating mechanism. Much of the literature on UPB relies on social exchange theory and reciprocity-based explanations (R. Ahmad et al., 2024). We extend this work, applying the COR theory. We conceptualize ostracism as a form of resource loss and UPB as a compensatory response. This perspective broadens the theoretical lens, offering a more dynamic explanation of the reason that employees engage in unethical behavior under social strain.

Third, this study introduces underdog expectations as a key boundary condition. The findings obtained here show that employees do not respond to ostracism in a uniform fashion. Instead, their reactions depend on how they interpret their social standing. That is, employees who feel underestimated are more likely to translate ostracism into UPB. This insight deepens the understanding of the motivational processes that underlie unethical behavior and highlights the role of individual differences in producing responses to adverse workplace experiences. Taken together, these contributions integrate leader traits, resource-based theory, and individual differences into a unified framework. This framework provides a more precise and context-sensitive explanation of UPB, offering a strong foundation for future studies.

### 5.2. Practical Implications

These findings have important implications for leaders and organizations. First, leader narcissistic rivalry was positively associated with increased perceptions of workplace ostracism, indicating that interpersonal aspects of leadership can play a crucial role in the shaping of employees’ social experiences in the workplace. Although narcissistic traits can manifest as confidence and charisma, organizations should carefully consider the potential interpersonal drawbacks of such characteristics when contemplating making leadership-related decisions. More broadly, our findings underscore the necessity of leadership assessment and development strategies to promote inclusive interpersonal behaviors and minimize the risk of employee exclusion.

Second, our findings indicate that workplace ostracism could serve as a mechanism that links leader narcissistic rivalry to UPB. This highlights the significance of employees’ social experiences in understanding how leader characteristics could influence unethical workplace behavior. Thus, organizations should pay close attention to exclusionary dynamics and proactively foster inclusive work environments. It can be particularly effective to encourage leadership practices promoting inclusion, trust, and positive interpersonal relationships. Prior research supports the value of leadership approaches, such as inclusive and authentic leadership, in mitigating workplace ostracism and its negative consequences (Jang & Chen, 2022; Y. Yang et al., 2026). By addressing ostracism, organizations can better protect employee well-being, create a more ethical, inclusive workplace, and reduce the likelihood of UPB.

Finally, underdog expectations have a significant role to play in shaping employees’ responses to ostracism. While these expectations are frequently regarded as sources of motivation and resilience, this study demonstrates that they could also yield unintended negative consequences. In particular, employees who had stronger underdog expectations were more likely to engage in UPB in response to ostracism. This underscores the necessity of a nuanced understanding of both the advantageous and potentially detrimental effects of underdog expectations within organizational settings. Organizations should be mindful that motivational beliefs stemming from perceived disadvantage do not universally lead to positive outcomes (Nurmohamed, 2020), in particular not under conditions of social exclusion. Drawing on prior research, this study suggests that providing constructive feedback and cultivating a supportive work environment may enhance employees’ sense of competence and belonging, thereby mitigating harmful underdog perceptions (Loi et al., 2022). Further, previous studies suggest that reinforcing ethical norms, rewarding legitimate performance, and modeling ethical behavior can be effective strategies for discouraging unethical practices, such as UPB (Yan et al., 2024).

### 5.3. Limitations and Future Research Directions

This study presents several limitations that suggest directions for future research. First, this investigation relied upon single-source data. Although self-reports are appropriate and widely used for sensitive constructs such as UPB and perception of leader narcissistic rivalry (R. Ahmad et al., 2024; Carpenter et al., 2017; Fehn & Schütz, 2021), the reliance on a single source prompts concerns regarding CMB (Podsakoff et al., 2024). We sought to mitigate this risk using a three-wave, time-lagged design, and the statistical analysis indicated that CMB was not a serious threat. However, these procedures cannot completely eliminate the potential for CMB. In addition, although the study achieved a satisfactory overall response rate, a formal assessment of non-response bias was not conducted, and its potential influence, therefore, could not be ruled out entirely. Therefore, future research should employ multisource designs, such as integrating employee, supervisor, and peer reports, and assess potential response-related biases to strengthen causal inferences and the robustness of the findings.

Second, we examined workplace ostracism as a general perception without distinguishing its source. This limits the precision of the study. Prior research shows that ostracism from supervisors and coworkers could produce distinct and interacting effects (Ferris et al., 2008; Z. Wang et al., 2021). Future studies should examine workplace ostracism through distinguishing the sources to determine whether leader-driven ostracism differs from peer-driven exclusion in the prediction of UPB. This work would refine the proposed mechanism and improve the theoretical specificity. Further, while the proposed relationships were supported, the models exhibited only modest explanatory power. This suggests that workplace ostracism and UPB are likely affected by individual, leadership, and organizational factors that were not addressed in this study. Future research should examine other relevant antecedents, including abusive supervision, ethical climate, and related contextual variables, to provide a more comprehensive understanding of these outcomes.

Third, this study incorporates data from employees in a single national context, in particular, that of South Korea, which may restrict the generalizability of the findings. The relationships that were examined were found to be supported in this sample, but their relevance to other countries and organizational settings remains uncertain. Future research should replicate and expand this model across various national contexts and samples to enhance the generalizability of the results.

## 6. Conclusions

Drawing on COR theory, this study examines whether, how, and when leader narcissistic rivalry can drive employees’ UPB. The study findings show that leader narcissistic rivalry can increase workplace ostracism, which in turn promotes UPB. In other words, ostracism operates as a mechanism that links leader narcissistic rivalry to unethical behavior. Employees who feel excluded experience a loss of critical relational resources. They respond by engaging in UPB to restore inclusion, regain status, and signal loyalty to the organization. This pattern supports a resource-based explanation of the reasons that harmful leader traits can produce unethical outcomes. However, this effect is not uniform. Underdog expectations amplify the ostracism of the UPB relationship. Where employees believe that others underestimate them, their exclusion becomes more psychologically salient. As a result, they become more likely to engage in UPB to prove their value and reestablish their standing. Overall, this study indicates the importance of leader personality, relational experiences, and individual differences in the shaping of unethical pro-organizational behavior. It indicates that narcissistic rivalry does not operate in isolation; rather, it triggers interpersonal dynamics that deplete resources and motivate compensatory behavior. We encourage future researchers to further examine how leader traits interact with workplace experiences and employee mindsets to influence ethically questionable behavior.

## Figures and Tables

**Table 1:** Confirmatory factor analysis results.

Model	Factors	χ^2^	df	χ^2^/df	CFI	TLI	RMSEA
One-factor model	F1 + F2 + F3 + F4	3817.633	209	18.266	0.543	0.495	0.213
Two-factor model	F1, F2 + F3 + F4	3076.506	208	14.791	0.637	0.596	0.190
Three-factor model	F1, F2, F3 + F4	1172.227	206	5.690	0.878	0.863	0.111
Four-factor model	F1, F2, F3, F4	469.191	203	2.311	0.966	0.962	0.059
Five-factor model	MFLV, F1, F2, F3, F4	467.221	201	2.324	0.966	0.961	0.059

Notes. F1 = leader narcissistic rivalry; F2 = workplace ostracism; F3 = underdog expectations; F4 = unethical pro-organizational behavior; MFLV = method factor latent variable.

**Table 2:** Means, standard deviation, and correlation.

Variable		M	SD	1	2	3	4	5	6	7	8	9
1.	Sex	0.64	0.481									
2.	Age	2.74	1.253	0.061								
3.	Education	2.38	0.562	−0.010	−0.058							
4.	Tenure	2.67	1.042	−0.080	0.082	−0.155 **						
5.	Leader Narcissistic Admiration	3.26	1.127	−0.013	−0.009	0.063	−0.056					
6.	Leader Narcissistic Rivalry	3.18	1.273	0.045	0.018	0.043	−0.010	0.498 **	(0.856)			
7.	Workplace Ostracism	3.32	1.216	0.015	0.053	0.013	−0.063	0.192 **	0.215 **	(0.860)		
8.	Underdog Expectations	3.71	1.255	0.023	0.152 **	−0.124 *	0.046	0.061	0.115 *	0.181 **	(0.862)	
9.	UPB	3.31	1.249	0.126 *	0.056	0.000	−0.011	0.077	0.103 *	0.194 **	0.123 *	(0.871)

Notes. * *p* < 0.05, ** *p* < 0.01; UPB = unethical pro-organizational behavior. The square root of AVE appears in parentheses.

**Table 3:** Regression analysis results.

Variables	Workplace Ostracism		Unethical Pro-Organizational Behavior	
	β	SE	β	SE
Control variables				
Sex	−0.003	0.128	0.309 *	0.132
Age	0.053	0.049	0.038	0.050
Education	−0.010	0.110	−0.001	0.113
Tenure	−0.075	0.060	0.009	0.062
Leader Narcissistic Admiration	0.054	0.091	−0.003	0.093
Independent variable				
Leader Narcissistic Rivalry	0.165 *	0.080	0.059	0.083
Mediating variable				
Workplace Ostracism			0.184 ***	0.053
R^2^	0.054		0.058	
adj. R^2^	0.039		0.040	
F	3.561 **		3.279 **	

Notes. * *p* < 0.05, ** *p* < 0.01, *** *p* < 0.001.

**Table 4:** Results of the mediating analysis.

Paths	Effect	SE	95% CI	
			LLCI	ULCI
Total effect				
LNR → UPB	0.090	0.083	−0.074	0.254
Direct effect				
LNR → UPB	0.059	0.083	−0.103	0.221
Indirect effect				
LNR → Workplace Ostracism → UPB	0.030	0.017	0.003	0.069

Notes. LNR = leader narcissistic rivalry; UPB = unethical pro-organizational behavior.

**Table 5:** Results of the moderation analysis.

Variables	Effect	SE	t-Value	95% CI	
				LLCI	ULCI
Workplace Ostracism	0.198	0.052	3.824 ***	0.096	0.300
Underdog Expectations	0.109	0.051	2.128 *	0.008	0.209
Workplace Ostracism × Underdog Expectations	0.146	0.041	3.620 ***	0.067	0.225
Levels of Underdog Expectations					
−1 SD	0.015	0.069	0.221	−0.121	0.151
Mean	0.198	0.052	3.824 ***	0.096	0.300
+1 SD	0.381	0.076	5.039 ***	0.232	0.529

Notes. * *p* < 0.05, *** *p* < 0.001.

## Data Availability

The data that support the findings of this study are available from the corresponding author upon reasonable request.

## Abbreviations

The following abbreviations are used in this manuscript:

COR	Conservation of Resources
LNR	Leader Narcissistic Rivalry
NARC	Narcissistic Admiration and Rivalry Concept
UPB	Unethical Pro-organizational Behavior
CFA	Confirmatory Factor Analysis
CMB	Common Method Bias
